# Opportunities: Source of synergy, or of conflict? Positioning of creative industry actors within a European Capital of Culture Project

**DOI:** 10.1371/journal.pone.0274093

**Published:** 2022-09-13

**Authors:** Nicolae Popa, Alexandru Dragan, Alexandra-Camelia Marian-Potra, Marius Lupsa Matichescu

**Affiliations:** 1 Faculty of Chemistry, Biology, Geography, Department of Geography, West University of Timișoara, Timișoara, Romania; 2 Faculty of Sociology and Psychology, Department of Sociology, West University of Timișoara, Timișoara, Romania; Shenzhen University, CHINA

## Abstract

The aim of this article is to analyze the positioning and interactions of the principal actors in the creative industries, in particular their relations with the structures tasked with implementing the Timișoara European Capital of Culture 2021 project, and the press coverage of the developing project. We started from the hypothesis that such an event stimulates the creative industries, but also induces tensions, which can degenerate into blockages. Quantitative and qualitative methods (20 observation sheets; 19 interviews and 227 press articles) combine to portray the dynamics and effectiveness of the city’s creative spaces and the role actors play in local territorial co-production, the press analysis providing additional data. The article supplements previous literature on politico-territorial arenas by spotlighting spatio-temporal reconfigurations that spark synergies and tensions within an urban environment with a long-standing multicultural tradition. The results of our research highlight, on the one hand, the tensions and blockages in the public sector in the implementation of the project, marked by political rivalries at various levels, and on the other hand the critical attitude and difficulties in coagulating the energies of the local creative cultural sector. The press analysis reflects a complex and contradictory reality, with both positive aspects (cultural events, dynamics, public involvement, etc.) and negative ones (leadership, dissensions, underfunding). We demonstrate the response of creative industries facing a major cultural project with great potential for stimulating urban development but whose success may be threatened by weaknesses in leadership, in participating governance, and in local civic involvement.

## Introduction

Opportunities have to do with circumstances, yet exploiting them is contingent on the coming-together of a wider spectrum of factors and contexts that give life to systems both from inside and from outside. Within territorial units, studies of the theory of opportunity relate this idea to equality/inequality and to self-determination. The distinction between morally acceptable and unacceptable inequalities, a major contribution of egalitarian philosophy during the past five decades, has now passed into social practice [1] Economists normally evaluate opportunity via results obtained, without taking account of the role of the personal choices of individuals/groups or the situations in which these were made, results which do or do not provide equality of opportunity [2].

Starting from the premise of exploiting opportunity through providing equal chances, this article focuses on the processes of cohesion-dissuasion that spring from perceptions of a system’s ability to provide equal chances and properly exploit opportunity. Winning the European Commission-granted title of a European Capital of Culture (ECoC) is a rare opportunity for a city to make its mark–so rare that in the almost forty years of its existence only Luxembourg has held it twice.

Therefore, the *principal objective* of our article, flowing from this outlining of the issues, is to identify the actions taken by the main actors involved in the creative industries, their interactions with the structures responsible for implementing the ECoC TM2021 project, and the way all this has been reflected in the press.

To do this, we started out from the *general hypothesis (I*.*0)* that winning the title of European Capital of Culture constitutes a stimulus for the creative industries to express themselves, but that their flourishing depends on different factors of training and support for entrepreneurship and creative initiatives.

We have added two further *specific hypotheses*, as follows: the implementation of the ECoC TM2021 programme has increased both cohesion and conflict between local and national actors, due to differences of vision between the principal actors and to competition to access resources (I.1); local convergences and divergences are felt by actors involved in culture and in the creative industries and are reflected in the mass media in a nuanced way, depending on their experiences and affiliations (I.2).

## Creative industries in the ECoC context

All ECoC projects aim to promote local identity and urban regeneration through culture [3], creating an optimal context for dialogue, reflection and the exchange of best practice [4], and for the promotion of cultural diversity, intercultural dialogue and the encouragement of creativity [5]. Innovation via creativity within urban governance is one of the principal coordinates of ECoC projects, and being a creative city is frequently associated with ECoC status [6]. Schlesinger [7] regards creativity as a “hegemonic” term which, when present in cultural policies, guarantees success in achieving the objectives of an ECoC project [3,8].

While the importance of the creative sector is frequently invoked in European projects, this can be a very challenging sector for local government actors to deal with [9]. Its functioning is largely dependent on government policies and the availability of public funding, with the State playing the role of initiator and coordinator of measures that can impact it [10]. The expression *creative economy* reflects the relationship of (inter)dependence between several kinds of actors: creative economy initiators, public sector decision-makers, NGOs [11], and the public as creator and consumer.

At the same time, the creative economy informs about the subjective experience and tensions associated with creative work. Such tensions intensify when there are perceived dysfunctionalities in the socio-political context in which creative activities are taking place [12].

Holding large-scale cultural events has a multivariate spatio-temporal impact on a community: it creates an *image builder* and stimulates urban regeneration, social cohesion [13], and the consolidation of infrastructural investment [14]. In this context, the starting hypothesis of *social exchange theory* [15] is that a group of individuals will wish to involve themselves in a process only if they will derive benefits from transactions taking place during it–a hypothesis tested at many large-scale events (the Olympic Games, the SuperBowl, ECoC2017 etc.). In other words, when the “costs” of relational development between actors outweigh the benefits, the relationship is likely to deteriorate or even end [16], the global project failing. The results identify four kinds of actors: (1) those who become involved and who have a positive perception of the role of ECoC status in promoting tourism (2) pragmatic people who are directly involved in the event but have a negative perception of ECoC (3) those who do not become involved yet have a positive perception and (4) non-involved people with a negative perception [14]. Consequently, *social exchange* is more than a simple cost-benefit trade- off; it is rather the sum of structural mobilisations in a local society [17]), in which coordination and cohesion are vital [18] if damage of the “struggle for opportunities” kind is to be kept to a minimum.

The operationalization of social exchange theory can be seen in this article both in the sense of commitment, respectively the extent to which actors engage in repeated exchanges with the same partners [19] and in terms of relational cohesion, based on emotions and daily satisfaction in a collective partnership [20].

Major cultural projects forge the mechanisms of social innovation at the local level [21]: we see (re)configurations of actors, new development strategies and new spaces for cultural display. Nevertheless, these frequently generate competing ideas, rivalries and conflicts, particularly when support structures, leaders or financial resources are lacking. However, conflicts may also be a stimulus to innovation [22], since they disturb routines and cause fissures in established patterns [23], which risks irritating those actors who see their locally accepted structures and achievements threatened by the process of “creative destruction” [7].

Cohesion among action groups and the effective functioning of the mechanisms through which they attain their goals depend on a number of factors including shared motivation [24,25] and the creativity of those involved and its appropriateness for the goals in view [26–28]. Disagreements and frictions arise from incompatibilities and from divergences in perception, expectations or opinion [29–31]. The likelihood of conflict is greater in heterogeneous than in homogeneous groups [32] because of the diversity and social distance between members. In heterogeneous groups, equality of chances is especially vital to ensure involvement and cohesion.

The choice of Timisoara as a case study for this paper is motivated by the following reasons:

- Timisoara is the largest and most dynamic urban agglomeration in western Romania, near Hungary and Serbia, with over 430 thousand inhabitants and more than 40 thousand students in universities with specializations in culture, art and IT, well integrated into international circuits;
- the city has long traditions of multiculturalism and intercultural dialogue, which have stimulated its European openness and cultural-creative initiatives;
- it has a typical Central European architectural heritage, concentrated in its four historic districts, attractive for cultural events and creative initiatives;
- it concentrates many industrial spaces from the first and second industrial revolution, some abandoned, which can be revalued as spaces of cultural experiment and creativity;
- in 2016, Timisoara was rewarded European Capital of Culture for 2021, after a tough competition with 14 other cities in Romania, which increased its national and international visibility, but also the public expectations related to the capitalization of this title.

The submission package was conceived and supported by the Timisoara 2021 Association (TM2021) together with Timisoara City Hall (PMT); these bodies also took responsibility for implementing the programme approved by the European Commission. This article throws light on the extent to which the Municipality and the TM2021 Association succeeded in harnessing local resources, creating synergies in the cultural sphere, stimulating the creative industries and setting in motion processes of urban regeneration undertaken in the BidBook.

To this end we asked ourselves a number of questions which have shaped the *issues addressed in the research*. To what extent are the actors most targeted by/involved in the project rising to the expectations of the Timișoara community and exploiting this opportunity by their actions? Does the city have quality leadership that can ensure cooperation between relevant competences and initiatives? When synergies arise, what mechanisms generate them? As for tensions and discouraging rivalries, are they due to failure to guarantee equality of opportunity, or to other factors and circumstances? Do these synergies and rivalries have more to do with individual/institutional responsibilities, or with chance [33]? What conclusions may be drawn from analysing the discourse used by those involved, above all those in the creative industries, and by studying press articles?

## Methodology

**Fig 1:**
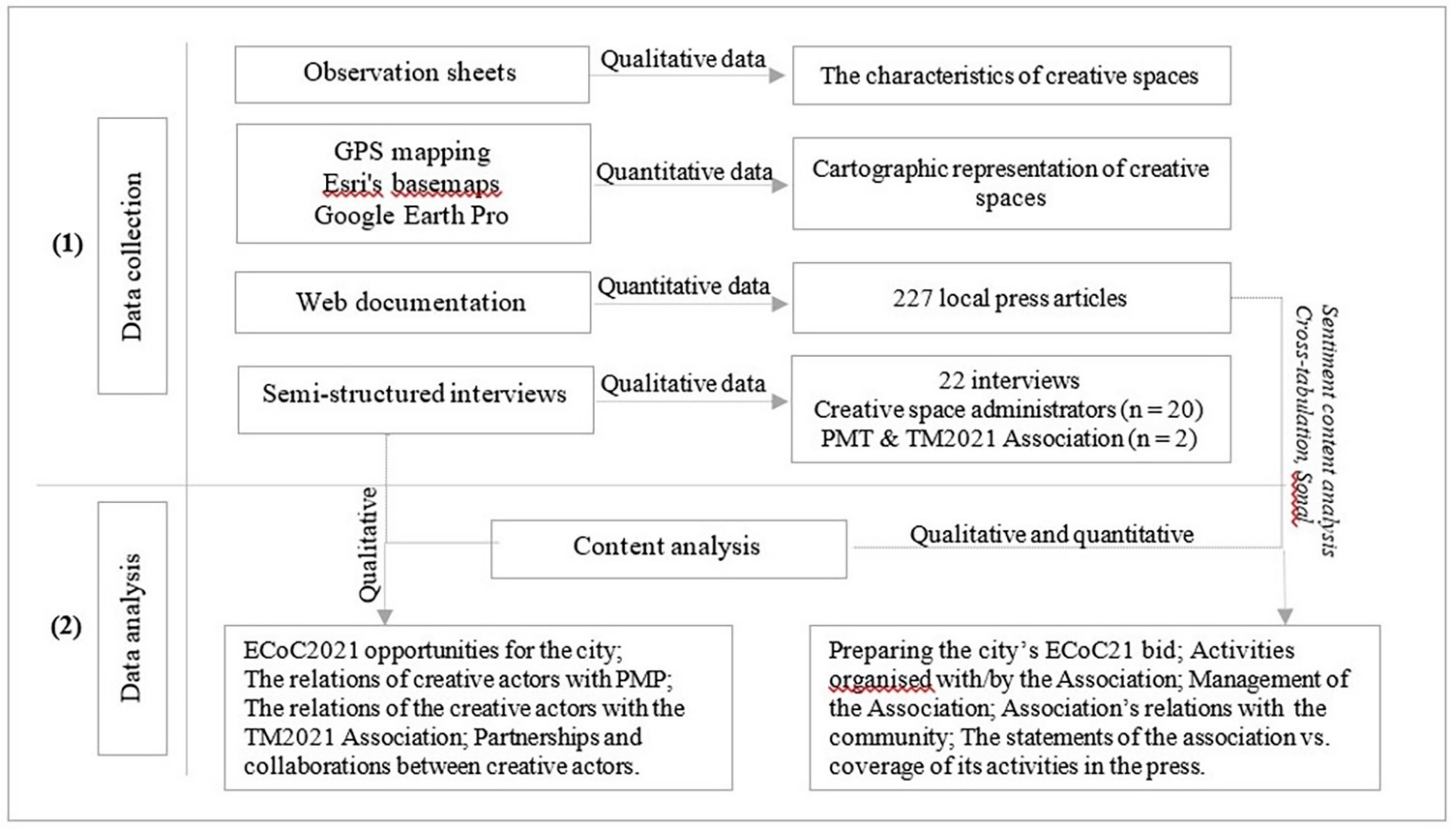
Design methodology flow chart.

To clarify the issues being discussed and test the hypotheses proposed, we pursued two main methodological directions: (1)obtaining quantitative and qualitative information regarding the dynamics and effectiveness of use of Timișoara’s creative spaces by completing observation sheets, GPS mapping of sites, applying semi-structured interviews and identifying relevant press articles; (2)data processing and analysis through cartographic representation of creative spaces and carrying out a content analysis of interviews with actors using them, correlating this with information provided by those responsible for the ECoC TM2021 project–a procedure mirrored by analysis of the press articles (Fig 1).

**Fig 2:**
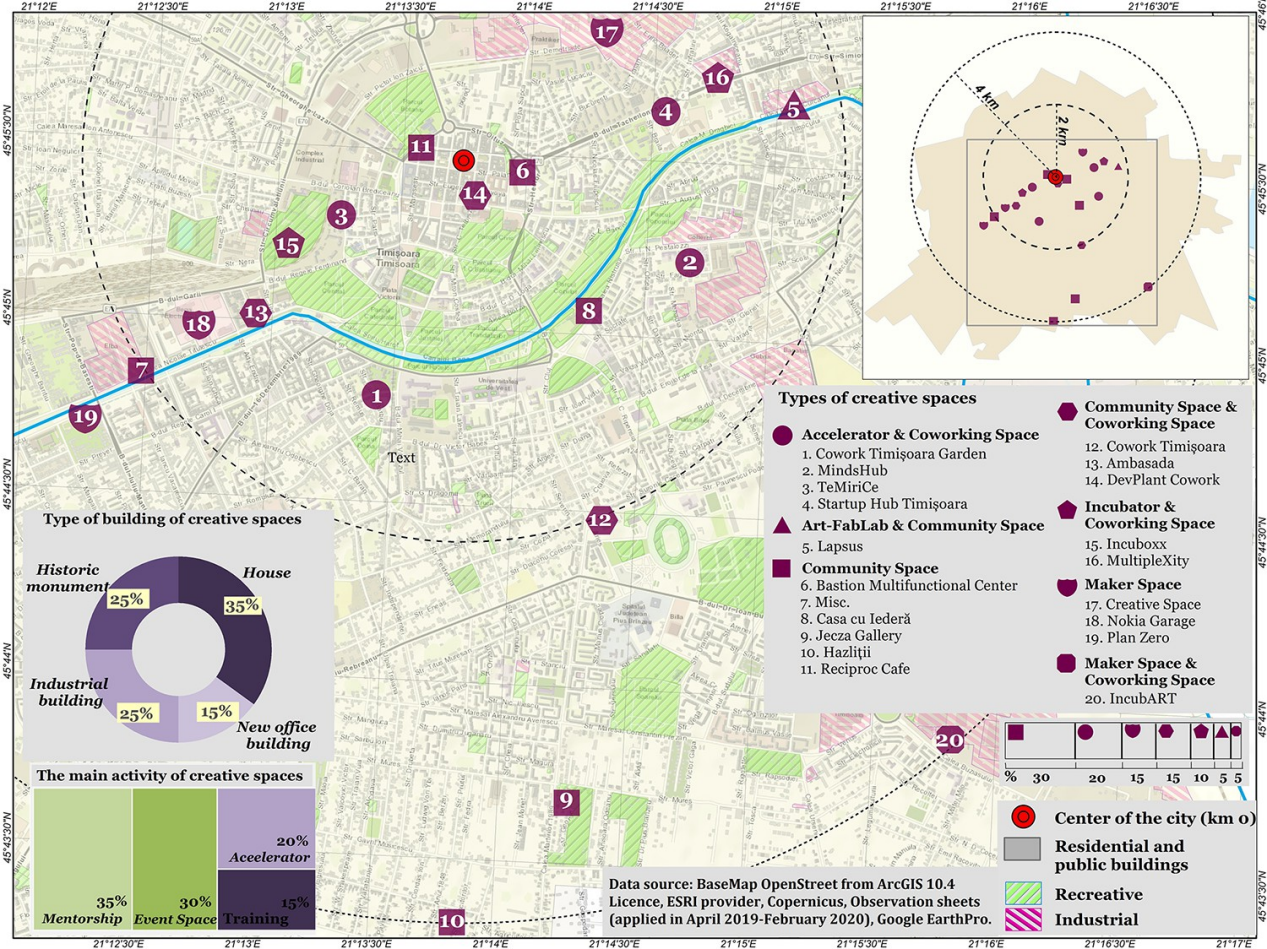
Sampling locations of creative spaces in Timișoara.

The use of the observation sheet facilitated the collection of field information regarding the accessibility and physical characteristics of creative spaces and the specific nature of the activities carried out there. The sheets were completed between April 2019 and February 2020, with 20 such spaces in Timișoara being mapped. The location of each identified space was based on geographical coordinates using GPS. The data on the features observed in the field were processed cartographically with ArcGIS 10.4 software and rendered as a map (Fig 2). The OpenStreetMap basemap in ArcGIS, provided as open data by ESRI (the developer of ArcGis software), was used to create the map. After mapping the creative spaces in the field, the following were applied qualitative methods [34]. Thus, 2 semi-structured interview grids were developed, one for *managers/administrators of creative spaces* and second for *those in charge of implementing the ECoC TM2021 programme* (the TM2021 Association and City Hall).

The next step was the analysis of articles from the Timișoara press dealing with subjects connected with the Timișoara ECoC2021 project. The digital versions of three press organs (*Renașterea Bănățeană*, *Opinia Timișoarei* and *Tion*) were used, this selection being made on the basis of experience and readership (continuity/regularity of publication, number of viewings) [35]. Articles were consulted for two time periods, the first while Timișoara’s application was being written (2015 and 2016) and the second after it had won the title (2017-March 2020), with a total of 227 articles being identified.

Data extracted from the interviews with creative space administrators were qualitatively analyzed. Thus, they were divided into 8 categories [36], with an emphasis on the strengths, weaknesses, financial resources, collaborative relationships and future prospects of each space. The content of the interviews with leading representatives of the two structures involved in implementing the ECoC TM2021 project, City Hall and TM2021, was grouped into three categories: *preparing the title bid and the impact of winning*, *the outworking of the project*, and *creative spaces in the context of the staging of the project*. The data was studied separately for each category of respondents, and in complementary (the views of creative actors and those of representatives of ECoC TM2021), and comparative (the views of creative actors *versus* those of representatives of ECoC TM2021) ways, depending on the scope pursued.

Press articles were classified into 4 categories for qualitative analysis: preparing the city’s ECoC TM21 bid, activities organised by the Association or in conjunction with it, the management of the Association, and the Association’s relations with the socio-economic and political-administrative environment. These categories were then analysed according to emotional content (*sentiment content analysis*) using three codes, positive, neutral and negative [37]. Procedurally, these codes have been correlated with the news content through cross-tabulation analysis (combined analysis) [38,39].

The Sonal program [40] was used to analyse comparatively the interview content applied to the TM2021 Association and the titles of the press articles. The press titles and the interview content were both transformed into series of quantifiable data [41], which produced 1153 unique words in the press titles and 1413 in the interview. Those words were divided into thematic classes according to their meaning and meaning in the sentence [42].

## Results and discussion

### Timișoara, an environment with potential for the proliferation of cultural spaces

This study has assessed Timișoara’s creative milieu through the lens of the 20 spaces identified on the ground. This sample reflects a growth in creative industries, the great majority of them new for the city, all alike pursuing the expansion/development of the local creative community and of synergies based on shared values, along with the encouragement of urban innovation. Our grouping of creative spaces into clusters (Fig 2) is based on the specific features recorded on the observation sheets, with their different functions allowing them to be matched to the grid of types of creative spaces suggested in the *Creative Community Space* report [43]. While Timișoara has five main kinds of creative spaces (Accelerator, Community Space, Coworking Space, Incubator and MakerSpace), the interactive nature of the activities carried out means that 11 of the sites mapped display a hybrid functioning model that combines two or more kinds of creative spaces [44].

The largest number of sites portion out their space between foundational creative activities and those of a coworking type (n = 10). The coworking component, which brings together independent professional creatives from a variety of fields (digital art, IT, design, consultancy in creative areas), is frequently seen as a way of maximising the cost-effectiveness of the running of the creative structure as a whole. A second category well represented is that of Community Spaces (n = 9), sites designed to strengthen social cohesion through creative-cultural events such as art exhibitions, talks, workshops, performance art and so on. Makerspaces give access to innovative equipment in the realms of digital art, robotics, woodworking and ceramics and provide full-time mentoring. Structures of the accelerator (n = 4) and incubator (n = 2) type, including coaching, mentoring, and courses for start-ups, are angled towards developing the local business community in the creative domain.

Studying the geographical distribution of creative spaces shows that they are concentrated in the central and inner areas of the city, within a two-km radius circle centered on its point zero (Piața Unirii). This gives them good access to major arteries and by implication to public transport too.

The majority of the creative spaces identified have appeared in the past ten years. They complement the traditional structures that constitute the cultural and creative offer of Timișoara, a city ranking third in Romania after Bucharest and Cluj-Napoca for its creative industries [45]. While their development has been potentiated by the local university environment, it also owes debts to the wider socio-economic milieu. Our research results demonstrate that the continuing success of creative initiatives is also conditional on the willingness of local government to encourage creativity and to involve the independent creative community in exploiting development opportunities.

### Public and associated structures, between aspirations, commitments and impasses

The principal actors involved in winning the title of ECoC and in the successful execution of the project are Timișoara City Hall (PMT) and the Timișoara 2021 Association (TM2021), with the former responsible for the investment and regulatory framework and the latter for implementing the agreed programme.

The representatives of City Hall and of the TM2021 Association view Timișoara’s winning of ECoC status through the lens of the benefits and problems this will bring for the city. It is the *positive aspects* that stand out in their discourse. The City Hall spokesperson cites benefits experienced by other ECoCs: increased visibility of the city in Europe and in the world; an increase in tourist numbers of 12–100% in the year concerned, with continuing growth in succeeding years; general economic benefits (each euro invested producing 8–10 euros in the local economy in the year concerned and following years). There is also an expectation of benefits specific to Timișoara that will flow from the setting-up and objectives of the project, with a focus on community consolidation and changed mentalities.

*”We are looking forward to the formation of a more active community generally*, *not merely exceptional individuals*, *with people being more involved in the life of the city*, *who see culture as contributing to the development process*. *[…] Even more important is the legacy of ECoC status*: *better city infrastructure (for culture*, *creative industries*, *businesses)*, *more innovative and creative people*, *and a strengthening of the inclusive nature of culture so that even the uninitiated can access it”* (DD, Deputy Mayor).

The TM2021 Association too sees the title primarily in terms of the opportunities it is creating and their long-term impact. Five opportunities are highlighted. The first has to do with the candidature period, when the city’s civic energy generated a shared agenda seen as a measure of long-term commitment. The second concerns the development of human resources, given that “*projects of this amplitude require growing the organisational capacities of those in charge*”. The title has also connected the city to international networks, particularly in the area of creative and independent cultural industries, thus potentially “*differentiating the city from one that relies on the classic formulae of its public cultural institutions*”. Two further opportunities are concerned with the infusion of capital (around 60,000,000 euros as initially proposed), which translates as a rehabilitated or constructed cultural infrastructure and a strengthening of the city’s tourist brand.

These opportunity-centric views (on both sides) confirm the social science theories where costs have negative values and rewards have positive values: Worth = Rewards—Costs [46]. In practice, the involvement of individuals in a large-scale project, even one that contributes to the common good, depends on the benefits obtained; the struggle for opportunities thus goes beyond individual principles and transforms actions into group positioning aimed at obtaining benefits (financial, political, reputational etc.).

The TM2021 Association spokespeople believe that the project *per se* will leave a useful tangible heritage (for example, an art, technology and experimental project centre) and above all that the investment in human resources and the philosophy of financing being focused on organisational empowerment create the premises for the assured future of creative cultural initiatives:

*”The fact that we are giving them* [i.e. the small creative initiatives] *a framework through which their interventions can be more numerous and more visible strengthens the links between these groups; by giving them greater exposure we are helping them impact the collective mentality”* (TM2021).

The striking feature of the narrative constructed by the representatives of City Hall and TM2021 in their interview responses is their almost obsessive repetition of the statement *“The title belongs to the city*!*”* The words, always strongly emphasised, occur in four of the City Hall representative’s 16 answers, and in contexts that reveal at least the following points: a wish to stress the role played by City Hall in preparing the BidBook and creating the conditions for winning the title; an awareness of City Hall’s responsibility for rolling out the programme, including through funding; a justification of a possible more directive future involvement of City Hall in the management of the Association, tasked with running the artistic programme described in the BidBook.

The self-perception and institutional image projected by City Hall are preponderantly positive regarding the municipality’s contribution both to the success of the bid and to its implementation, especially through its provision of financial support. Having in the BidBook pledged to contribute 50%, in the absence of mobilisation by the other public and private partners it had, up to the interview date, contributed *86% of the funds spent on the Timișoara2021 programme* (interview with Deputy Mayor).

The TM2021 interviewees too emphasised two points: that the project belongs to the city (almost all they said refers not to any particular institution but to Timișoara), and that it has long-term impact. This last consideration has strategic importance, with even a proposal that after 2021 the city should aim for membership of the UNESCO *Creative Cities Network*, principally so that the prospect of a new and challenging agenda could save it from post-event nostalgia [47].

Both bodies also mentioned some *disadvantages and tensions* occasioned by winning the title. The TM2021 representatives accounted for the intermittent nature of cultural programmes in Timișoara on three grounds: experience-related, legislative, and financial. The first refers to a failure by actors to grasp the fact that *“a cultural capital is not merely a source of funding for a short period […] or an arts festival but rather a programme of development and investment in human resources […] and in networks in the cultural and creative industries”*. These conflicting visions generated tensions between the many cultural actors, with *“an experimental-avantgarde group and a large conservative majority”* clearly discernible.

Secondly, a difficulty presented as all but insurmountable is the absence of a legal modality for directing funds to any organisation except a state institution, which compelled the Association to waste its energies applying “annually, tranche by tranche and pointlessly” for the money needed for the programme to survive. Only in January 2020, after three and a half years of constant pressure, was the legal instrument created that allowed the Association to be declared a public utility. *“Romania was not ready for this project; a former CEO for Krakow 2000 described precisely the same problems*! *It’s interesting to see where we are now in 2020*, *compared with 2000 in Poland*!*” (DD)*.

Thirdly, TM2021’s financial difficulties are connected both with the legal ones and with the practical involvement of financers. Its chief partners are City Hall *“and this is natural*, *because the city wanted this and it is reasonable for it to make the largest investment”*, followed by the County Council and the Ministry of Culture. So far, the business community has been relatively passive financially regarding TM2021, with only infrastructure preparations (particularly in the hospitality industry) apparently being made; their financing of cultural projects is one-off in nature and often unconnected with TM2021. The absence of substantial funding and of financial predictability has created organisational instability within the Association, since it struggles to determine its human resources policy. A further consequence has been a decline in its partners’ (particularly those abroad) confidence in it. Despite all this, in 2019 TM2021 fulfilled its pre-arranged commitments by holding over 400 public events, involving 131,000 participants.

The City Hall representatives too also cited some financial issues, but from the perspective of the usefulness that different local budget allocations have for the city (“*Timișoara currently allocates 25% of its budget to culture*, *which some people regard as unjustified and unsustainable”–DD)*. Added to this is the stir that the importance of this event has created among the animators of local cultural life, with its consequences *(“it also matters how many actors remain in the battle to prepare and organise the ECoC and how much each contributes*!*”–DD)*. Following the international literature [2,48,49], this last problem may also be interpreted as reflecting the conflicted attitude of the local cultural-creative community, stemming from different perceptions of the equality/inequality of opportunities, initially or along the way.

### The public sector, a minefield of divergent interests and political rivalries?

The dysfunctions that exist in relations between the municipality and the other public institutions involved via the BidBook, the Government/Ministry of Culture and Timiș County Council (CJT), manifest themselves in a number of areas–financial-investment, attitude and involvement, organisational–both at national level and locally.

Regarding the parties’ financial involvement, the City Hall representatives believe they were insufficiently convincing and effective in their actions. The reasons are many. For the relationship with the Government/Ministry, chronic political instability at national level is blamed:”*It is hard for anything lasting to be settled given the nine different Ministers of Culture in 2016–2020*!*”* Feelings about the negative impact of political instability are heightened by the fact that in the other cultural capital in the region, Novi Sad, *“the Serbian government is the main financial supporter of the programme”* (DD).

Here, the difference in political color between the leadership of the city and of the county–a situation that frequently leads to impasses—would seem to have played a major role. The imminence of the local and general elections, planned for the second part of 2020, has increased party-line radicalisation.

We conclude that the diversity of parties involved is not always a source of dynamism; it can also produce conflicts and impasses [30–32]), as attested both by interviewee evidence and by other studies of ECoCs [50,51]. But conflicts can arise for more general reasons, not necessarily unconnected with political rivalries. They spring from the general variety of the local community, demographically transformed in the past 50 years [52] and diverse in its levels of education, rootedness in Timișoara life, and attachment to and confidence in city projects [53]. This partially explains why *“there were plenty of Timișoara people who predicted and gave reasons for (wanting it to happen*?*) the failure of the bid*, *even just a few days before the final decision”* (DD).

Tensions, perceived locally as dysfunctions, may also catalyse innovation [22], since they stimulate “creative destruction” [7]. Some established Timișoara creatives saw their local prestige threatened, which irritated them and made them react negatively and become vehement opponents. “*There have been and remain conflicts generated by culture people*, *some even leaders of Timișoara culture*, *who did not take part in developing the project and so do not know*, *understand or accept it”* (DD). As in other ECoCs, the tensions originated in the preparation period and erupted immediately after the victory, whether to justify a lack of confidence displayed along the way or, contrariwise, from frustration that the on lookers/opponents had lost the opportunity to be leading lights in an event involving so many interests.

However, the municipality sees the angst that accompanied the Timișoara ECoC2021 programme as having only minor wider reverberations and thus little potential to damage the domestic and international appeal of the events programmed, the target year and subsequently. *Externally*, *Timișoara’s image was little affected*. *Even in the rest of Romania the image of the city and the programme is better than in Timișoara”* (DD).

The local authorities, however, have different concerns, which may be structured in a few points. The first point was long concealed by the Municipality in an attempt to justify its decision not to meddle in the work of the Association, *so as not to be accused of political machinations*! The need for involvement has, however, been acknowledged with increasing frequency during the past year, as the deadline approaches.

The second point links with the first, since some activities and events held as part of the programme did not draw the expected audience. A deficient cultural grounding and poor local cooperation, together with contradictory discourse from different actors, created a feeling that the programme was too ambitious for our local resources, or inappropriate, or that the events had not been thoroughly prepared for, “*for lack of anyone to prepare them*. *[…] More careful Town Hall involvement could have averted some of the unresolved conflicts and the loss of valuable resources from the independent cultural community” (DD)*.

If we exclude the predicted catastrophic effects of the Covid19 pandemic, the most serious cause for concern would seem to be the limited strength and involvement of the local creative milieu, something felt both by the local authority representatives and by some owners of creative spaces and/or creative industry animators: *“there are not many proposals coming from the creative industries*! *By contrast*, *their support needs are very diverse…”* (DD). This view contradicts to some extent other statements made in the interview, based on official statistics which rank Timișoara third in Romania for its creative industries *[45].*

### Critical perspectives from the Timișoara creative community

The creative milieu is nuanced and often cautious in its perception of and behaviour towards the local authorities. While the City Hall representative cites the small number of initiatives they receive from the creative milieu, the latter’s representatives account for their caution by referencing the local authorities’ failings and their lack of interest in the relevant area.

*“The municipal authorities do not feel any duty to support promotors of culture*. *They ought to appreciate the fact that I have 1*,*300 square metres that I use in the service of the community*, *to increase the city’s prestige*, *when I could be using it*, *as others would*, *for the material benefit of my family*!*” (SJ*, *Triade Foundation)*.

The leaders of creative bodies that function independently of the local authorities state that their main motive for non-participation in projects shared with City Hall or publicly funded is to avoid bureaucratic procedures and the reams of justifying documentation the authorities require in exchange for sums that are usually modest and paid very late. “*We are hobbyists here and we don’t really have time to get mixed up in paperchases*!*”* (MD, Plan Zero). “*Institutions are slow to react*. *In the start-up area*, *speed is extremely important”* (ACM, Cowork Garden). However, focusing too strongly on passion rather than project can be a risk for organizations, leading to ineffective leadership or leadership incongruence [54].

Interviewees from bodies whose articles provide for them to become structures in partnership with the local authorities or which belong to the authorities and were set up using European funds, despite being privately administered (Incuboxx, Multiplexity), describe different weaknesses and dangers involving influence from the political and economic interests of pressure groups or of other politically engaged entrepreneurs. These people, from working with City Hall, are acutely conscious of the local authorities’ lack of vision and thoroughness in implementing coherent medium and long term plans in this area.

*”As for the collaboration with City Hall and the local council*, *some people there are helpful*, *but others throw spanners in the works […]*. *A danger I see is a possible increase in pressure from political/economic forces wanting to take over and change our functioning*, *costing us our neutrality and the equal opportunities for start-up initiatives to gain access to Incuboxx”* (FV, Incuboxx).

Other testimonies describe relationships that are formal and official but emphasise the public authorities’ role in validating the importance of a project/event: *Our relationship with the authorities is practically non-existent*, *except that they are invited to events* (RT, Start-up Hub).

A smaller number of interviewees state that the main problem is the local authorities’ lack of response to suggestions from the creative milieu *(“…we offered ideas and availability*, *but nothing happens; we have no effective collaboration either with the local authorities or with the TM2021 Association”–*FC, Garage Nokia). This chimes with the small number of independent initiatives coming to City Hall and thus also the perception that the local creative milieu is limited.

### Partnerships and collaborations: A creative milieu that is hesitant, or connected?

Our research demonstrates the reality of a degree of anemia in the local creative milieu. A number of local actors sense this. It is ascribed to *“the novelty of this domain in the central- eastern part of Europe”* (DD), the strengths of the Romanian educational system, “*traditionally focused on* [Ivy House]), to “*censorship by the consensus”*, *through which people with original ideas are discouraged from expressing them”* (AB, Lapsus), to “*poor handling of the ‘lid’ that domination by corporations puts on the city’s progress”* (DB, Multiplexity), to the *“not very intelligent”* tendency “*to buy events rather than growing local creative abilities”* (SJ, Triade Foundation)–all reflected in the paucity of those interested in involvement in the creative industries.

Some entrepreneurs voice still other, more general dissatisfactions connected with the lifestyle of a younger generation accustomed to the comforts of the consumer society. *“We are fully equipped as a makerspace*, *but people don’t come […]*. *People are comfortable*. *They don’t want to come and work at something but to take it away ready-made”* (AV, Creative Space). This sum of findings appeals to the background of reluctance to collaborate, mentioned by the actors interviewed, or to the lack of agreement or congruence of values [55,56] caused by different actors having different goals.

The chief problem facing Timișoara’s creative sector, as highlighted by the TM2021 Association, is the absence of strong organisations capable both of serving as a model and of coordinating major projects. Without these, the small initiatives “*lack capacity and are always dependent on financers”*; it is hard for them to escape from a vicious circle in which funding is “*project-oriented*, *not infrastructure- or organisational strengthening-oriented”*, with most projects being short-term. Nevertheless, the TM2021 representatives believe that Timișoara has a genuine chance of becoming a centre for the creative industries, since the ingredients are there: universities proficient in the IT and technology fields, human resources, implementation know-how, *“a tradition of experimentalism*, *multiculturalism*, *and the entrepreneurial spirit* […] *that border towns have*, *which give it at least mental openness”*, ideal ingredients for the new media arts and IT&Technology mix. In the absence of strong organizations, one solution would be to join forces, but poor communication between the parties or lack of mutual trust [57] limits the local creative sector.

An important role has been played by the concentration on training in IT in public education, especially at university level (UPT, UVT), then the arrival in Timișoara from the late 1990s onwards of branches of foreign or domestic capital IT companies (Alcatel, Siemens, Nokia, Atos, Continental, Movidius etc.), and EU funding for business incubators and technology parks (Incuboxx, PIT). This has produced a bubbling local environment, stimulating for young people, in which those who were children 10 years ago, being taught, generally for free, by the young ITers of that time, are now instructing (for free) and even providing financial support for children and young people who want to make a start in the digital area, including those open to creativity, art and culture.

A study of partnerships and collaborations set up by Timișoara creative spaces reveals a fascinating situation. Analysing the kinds of collaborations in operation, by distinguishing partnerships set up at local, national, regional, European and international levels.

This focus on local collaborations emerges from the fact that 11 of the 20 interviewees say they collaborate with local public institutions. Those mentioned are preponderantly Timișoara’s universities. Collaborating with universities means gaining expertise and creative young people from them. The *Te Miri Ce [You wonder what]* spokes person gives a revealing description: “*Members of the teaching staff are frequently invited to give talks as part of the events we hold”*. By contrast, in very few cases are creative space activities supported or staged in collaboration with City Hall.

Collaborations with local private companies are approximately as common as those with the universities, with 12 out of 20 creative spaces saying they have collaborations or partnerships with local firms. Such collaborations most often take the form of companies funding creative space projects, or of paying rent, as in the case of Incuboxx, which houses a number of start-ups initiated by local entrepreneurs. What is certain is that the relationship between local creative firms, particularly those just starting, and the business incubators is a very close one, with the latter being where many successful companies came into being. An example would be *123 Form Builder*, which has progressed from its inception in the Incuboxx creative space in Timișoara to being an internationally known company.

Partnerships with local NGOs are the most common form of collaboration entered into by the creative spaces we studied. No fewer than 14 of the 20 said that their work involved collaboration with these. There were various kinds of collaboration, from the hosting of meets (Creative Space, among others) to the provision of support for community interest projects (*Te Miri Ce*) or for those of an innovative or educational nature. Here the approach of *Start-up Hub* is revealing. Begun as a project designed to transfer good managerial practices to Timișoara companies and businessmen, over time its work was redirected towards education, with particular emphasis on training young people as programmers. This creative space thus became the birthplace of *Coder Dojo Timișoara*, an NGO which has up to now already started 19 nodes in the region, nodes in which those as young as seven can learn to code and program.

A smaller number of Timișoara’s creative spaces also have collaborations at national level, six of the 20 collaborating with similar spaces around the country. *Incuboxx Timișoara* has links with other incubators in Cluj and Bucharest and is also contributing to starting one in Brașov, while *Garage Nokia* is liaising with universities not only in Timișoara and the west of the country but also in Bucharest to produce a lifesize 3D-printed human robot. Collaborations at regional, European and international levels are on a similar scale. Five representatives say they have collaborations and partnerships at regional or European level, and only three outside Europe. The people at *Start-up Hub* have set up partnerships and events with partners in both Serbia (Belgrade and Novi Sad) and Hungary (Szeged), their aim being to hold events involving partners from all three countries. Creative spaces of a cultural kind, such as the *Triade Foundation* and *Ambasada*, and also *Garaj Nokia*, which is technical, are the most open to forming European and international links.

### The local press and the faces of Janus

The role of the press in forming opinion and mobilizing society, including in creating community coalitions, is an acknowledged one [58,59]. The press influences the way people understand, evaluate and act in a given situation and how they interpret their memories of it [60].

For our study of the press’s handling of the work of the TM2021 Association we used the online version of the main local newspapers: *Renașterea Bănățeană*, *Opinia Timișoarei* and *Tion* for the period January 2015-March 2020, identifying 227 articles.

**Fig 3:**
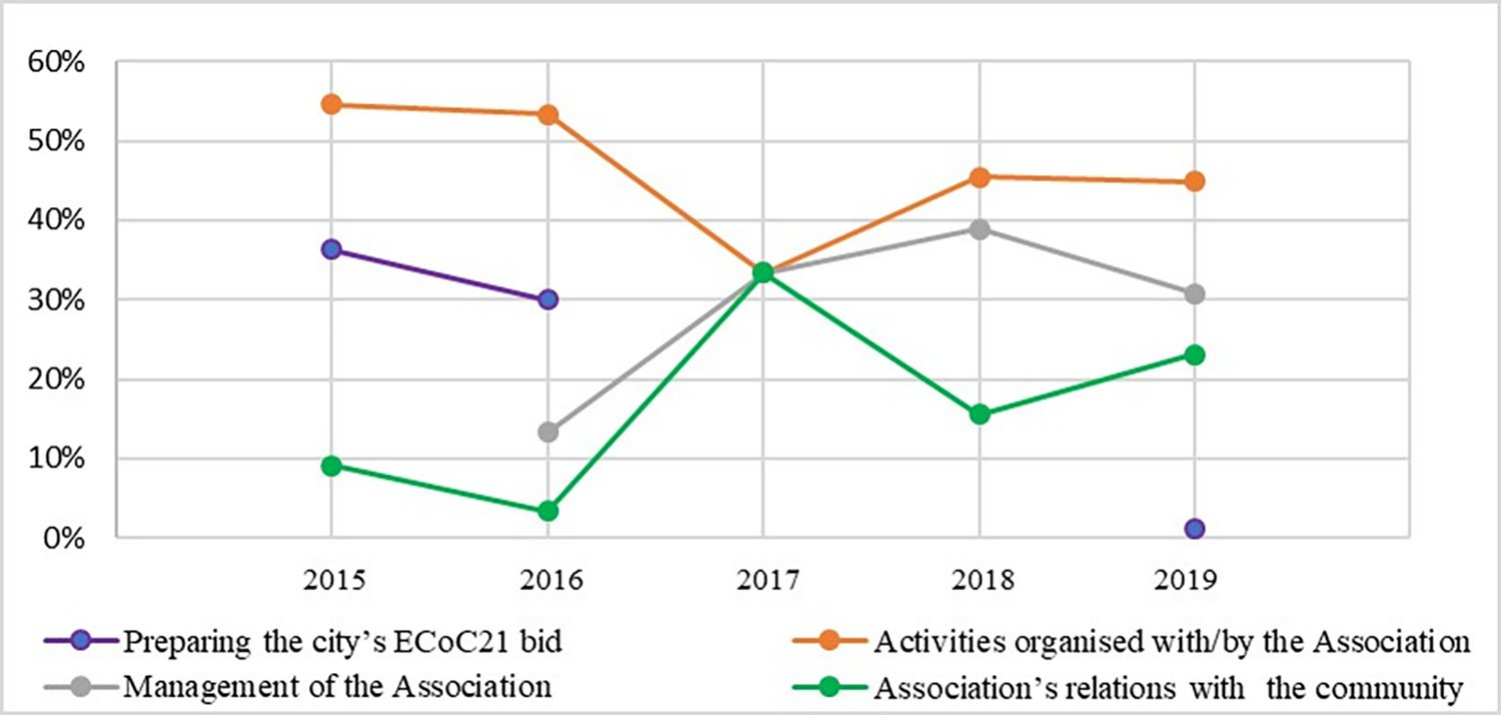
Thematic trends in the publication of press articles, 2015–2019.

Our content analysis of the press articles studied the coverage of activities and events connected with the Timișoara ECoC 2021 project (Fig 3). 43.2% of the articles identified from January 2015 to March 2020 dealt with cultural events. We observed a higher level of interest in this topic in the candidature period (53.3% in 2016), a constant level in 2017–19 (around 40%) and little interest (6.7%) in January-March 2020 –a period unsuited to outdoor cultural events. The management of the Association featured in 29.3% of the articles studied, the majority in the period 2017–19, with reports of delays and redirections in the allocation of funding for the project’s cultural activities, and of resignations from the TM2021 Association board. Articles covering the Association’s relations with socio-economic and political- administrative structures, which made up 21.4% of the coverage in the period studied, were higher in percentage terms in January-March 2020 (73.3%). The lowest percentage of articles (6.1%) dealt with events in 2015–16 in preparation for the bid.

The categories of subjects addressed in the 227 press articles were also analysed in relation to the direction of the news content. Analysis of the emotional content allowed it to be classed as positive, negative, or neutral. It was found that positive articles predominated (annual average 78.6%, with a high of 88.5% in 2018), while negative articles (averaging at 14.4%) reached their peak in 2020 (33.3%).

**Fig 4:**
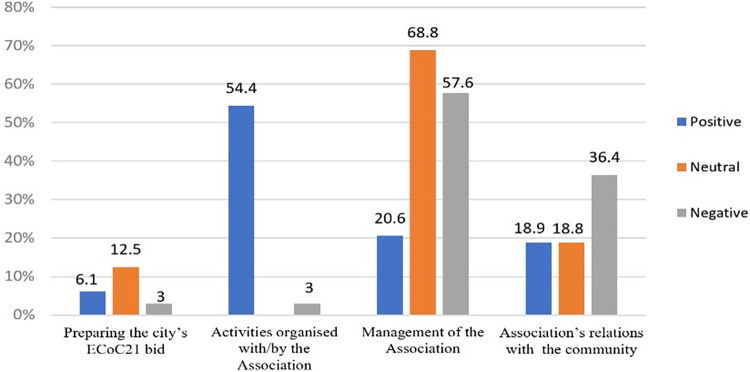
Type of subject versus content attitude.

Combined analysis of the two criteria–*types of subjects covered in the press and attitude taken in the articles*, coded as positive, negative, or neutral–brought to light the following facts (Fig 4): little information about the process of preparing the bid, but objective in its content and often linked to administrative details; a large number of reports of events run by the TM2021 Association, in appreciative press articles (we underline that the absence of neutral content may be a sign of a tendency for events to be over-praised in the press); an above average level of negative content in reports about the management of the TM2021 Association, with details detrimental to its image (losses of funding, resignations etc.), and a higher level of neutral content in the discourse of administrative and political decision-makers with regard to the management of the Association; the clearly negative tone of articles about the TM2021 Association’s relations with the public, regarded by the press as the most problematic area.

**Fig 5:**
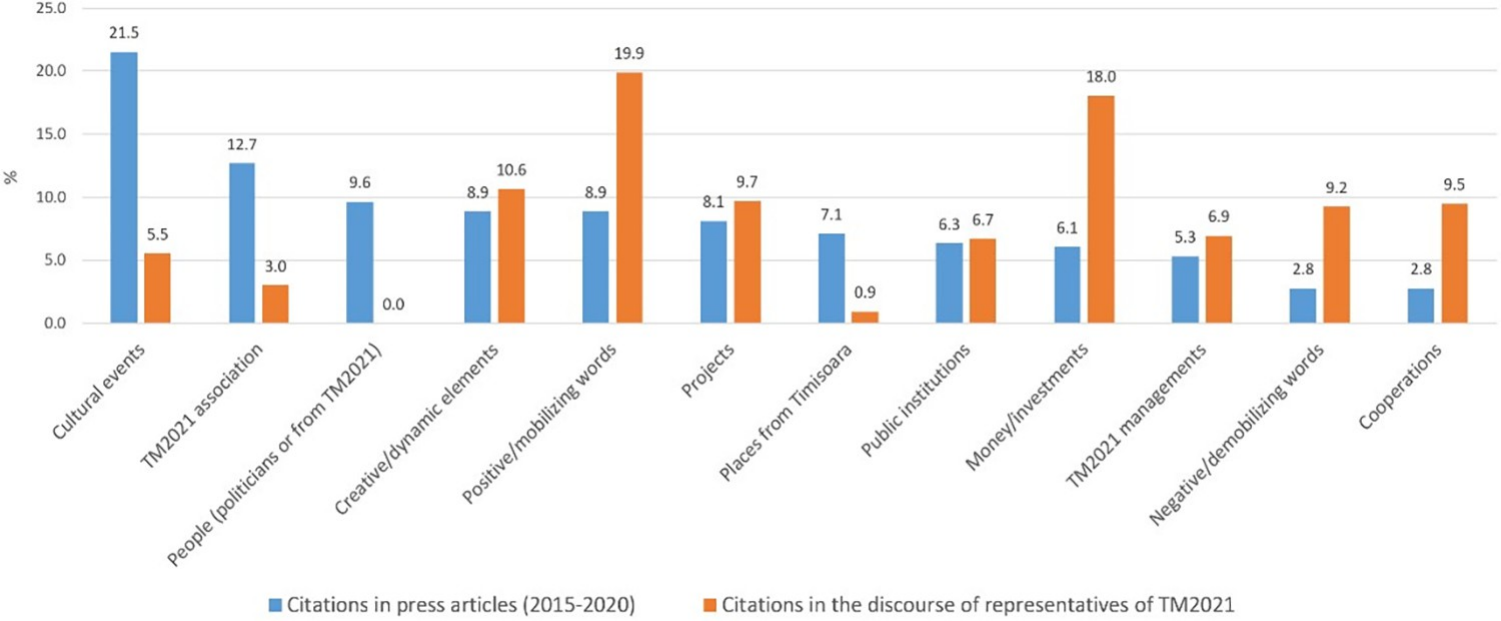
Percentages of thematic references concerned with the TM2021 project in titles of articles in the local press, 2015–2020, and in the discourse of representatives of the Association (interviewed March 2020).

For a cross-tabulated image [61] of the ECoC TM2021 phenomenon, we chose to compare the content of the entire corpus of 227 press article titles with the statements of the representatives of the TM2021 Association, interviewed in March 2020 (Fig 5).

In the local press article titles, 21.5% of words refer to cultural events held under the aegis of or in partnership with the TM2021 Association, 12.7% to the Association *per se*, and almost 10% to public figures (mainly in the sphere of local and national politics, but also in the management of the Association). The smallest numbers of references are to the Association’s collaborations and to discouraging factors (some 2.8%) and to its internal management (5.3%).

There is an obvious discrepancy between the press titles and the discourse of the representatives of the TM2021 Association interviewed, with the latter weighted strongly in two opposite directions: on the one hand, 19.9% of their words have a mobilising/positive contextual attitude *(it is possible*, *there is/are*, *we*, *have*, *development*, *spirit*, *openness*, *transparency*, *creation*, *successful*, *done*, *together*, *we must)*, but another 18% refer to concerning aspects, generally financial ones (money, euros, millions, budget, funding), which reflects a sense of impasse and frustration in the Association when the implementation of the programme is under consideration. Three further categories of words used by the TM2021 Association refer to new projects (9.7%), new collaborations (9.5%), and dysfunctionalities (9.2%) (*long*, *hard*, *need*, *problems*, *pressure*, *dissensions*, *we are losing*, *contestation*, *obliges*).

There are, nevertheless, two categories of words that both the press titles and the TM2021 Association make great use of: *new projects*, and references to *creativity/dynamism/new technologies*, which confirms the interpenetration of the idea of cultural capitals with that of cities of the future.

## Conclusion

This paper is part of the consolidation of the social exchange theory, drawing attention to the social and organisational fractures that appeared after the title bid succeeded. The involvement of individuals in a large-scale project, even one that contributes to the common good, continues to depend on the benefits derived: the struggle for opportunities thus overcomes individual principles and transforms actions into group positioning’s aimed at the gaining of benefits (financial, political, reputational etc.). Such contrasts are explained by Heckathorn [62] as a product of two factors: the explicit negotiation over social goods and the individual choices to abide by the terms of trade. He therefore argues that the exchange involves not only a negotiation of social goods, but also the game of a *prisoner’s dilemma* in fulfilling social obligations.

Our research results have allowed us to verify the starting hypotheses of this study and to clarify some points relating to the relationships between the dynamics of the phenomenon of creativity and the progress of the Timișoara ECoC2021 programme. While the rise of creative spaces in Timișoara was driven forward by the chance of winning the ECoC title, they drew their main support from European funding, accessible both to public authorities and to NGOs and private entities for human resources training, transfer of technology, and entrepreneurship development. Education, volunteering, community involvement and innovation catalysed one other and made significant contributions to the sustainability of the phenomenon, a point which confirms our general hypothesis (I.0). Consequently, the creative spaces, connected especially but not exclusively to the city’s IT community, became a self-sustaining phenomenon, independent of the contribution of or support from the local authorities or even from other major initiatives such as ECoC. The authorities are in most cases spectators of the creative phenomenon, while the latter’s involvement in the ECoC programme proved to be effective, particularly in the period when the project proposal was being conceived and written. Once the title had been won, the TM2021 Association made far fewer proposals to the creative milieu for the development of shared approaches.

The article’s contributions to an understanding of how the creative industries relate to the ECoC programme (l.1) and of its coverage in the mass media (l.2) are relevant, with results that confirm the hypotheses advanced. These also confirm the *social exchange theory* variables, by drawing attention to the social and organisational fractures that appeared after the title bid succeeded. The article also adds to the literature on politico-territorial arenas [63] by highlighting the reconfigurations that took place during the process of co-producing this great cultural project: the forging of innovations, the tracing of territorial connections and limits, the highlighting of strong points, but also the weaknesses of the local collectivity (inter-generational conflicts, the lack of leaders, and the damage caused by power struggles). The article contributes to the literature on creative spaces by confirming that these do not depend directly on one single city- level project but on multiple ingredients: leadership, the university sector, the availability of spaces, etc.

In 2020, immediately before the final stage of the ECoC2021 programme, all these challenges were intensified by an entirely unforeseen chance factor, the Covid19 pandemic, whose expected socio-economic impact is likely to be profoundly destabilising: *“We do not know how society in general will be affected*: *how movement will be restored*, *tourism*, *the desire for culture*, *what the priorities will be for people*, *the city*, *Romania… In fact right now we don’t even know what year it will say after Timișoara ECoC*: *2021*, *2022*, *or 2023*!*”* (Interview with DD, Deputy Mayor). Now we know: at the beginning of this year (2021), the European Commission carried over the title for Timișoara in 2023!.

## Supporting information

S1 File (XLSX)

S2 File (XLSX)
